# Touch and voice have different advantages in perceiving positive and negative emotions

**DOI:** 10.1177/20416695231160420

**Published:** 2023-03-21

**Authors:** Rika Oya, Akihiro Tanaka

**Affiliations:** Graduate School of Humanities and Sciences, Tokyo Woman's Christian University, Tokyo, Japan; 53347 Japan Society for the Promotion of Science, Tokyo, Japan; Department of Psychology, Tokyo Woman's Christian University, Tokyo, Japan

**Keywords:** emotion, perception, touch, voice

## Abstract

Previous research has revealed that several emotions can be perceived via touch. What advantages does touch have over other nonverbal communication channels? In our study, we compared the perception of emotions from touch with that from voice to examine the advantages of each channel at the emotional valence level. In our experiment, the encoder expressed 12 different emotions by touching the decoder's arm or uttering a syllable /e/, and the decoder judged the emotion. The results showed that the categorical average accuracy of negative emotions was higher for voice than for touch, whereas that of positive emotions was marginally higher for touch than for voice. These results suggest that different channels (touch and voice) have different advantages for the perception of positive and negative emotions.

Humans communicate emotions through various nonverbal cues such as face, voice, and touch. Many studies have investigated whether emotions expressed by nonverbal channels can be perceived or recognized. We define this process as the *perception* of emotions through nonverbal channels. Most studies have focused on the perception of emotions from face and voice, and have suggested that it is possible to perceive different emotions (Ekman, 1973; Scherer et al., 2011, as a review). For example, anger, fear, happiness, sadness, disgust, and surprise have been studied extensively to examine whether these can be perceived from facial expressions. Apart from the above six emotions, some complex emotions, such as contempt, awe, amusement, enthusiasm, and others, can also be perceived from vocal expressions (Simon-Thomas et al., 2009).

Other research has shown that anger, love, and gratitude can be perceived through touch (Hertenstein et al., 2006; Oya & Tanaka, 2022; Thompson & Hampton, 2011). Hertenstein et al. (2006) examined 12 emotions, including the above six, which have been well studied in the domain of facial expression, prosocial emotions, and self-focused emotions, to determine whether they can be perceived through touch. Based on the theory of the origins of cooperation and altruism (Frank, 2002; Sober, 2002), these authors assumed that it is possible to communicate prosocial emotions related to cooperation and altruism, such as love, gratitude, and sympathy, via nonverbal displays, particularly touch; results confirmed that anger, fear, disgust, and prosocial emotions were perceived. Moreover, Oya and Tanaka (2022) conducted almost the same experiment as Hertenstein et al. (2006) on Japanese dyads and examined the cultural similarities and specificities regarding the perception of emotion through touch. The results revealed that the accuracies for anger, love, and gratitude were above chance, suggesting that these emotions can be perceived from touch both in Japan and Western countries.

The literature suggests that emotions can be perceived through various nonverbal channels. However, to the best of our knowledge, the suitability of each channel for communicating specific emotions has not been examined sufficiently. It is difficult to compare channels because of differences in emotional categories and the methodology used in previous research. First, previous research has used different emotional categories for different channels. App et al. (2011) compared channels and demonstrated an association between an emotion and a particular nonverbal channel. In order to address the above issue, they selected 11 emotions due to their previously demonstrated use through one or more of the channels of interest: visual channel (body or face) and tactile channel (touch). They then divided these emotions into three types according to social function: social status, survival, and intimate relationships. The results suggest that channel preferences are related to the social functions associated with each emotion. Their classification provided important suggestions for understanding channel preferences. However, these authors did not examine the preferences based on the classification of emotional valence. It would also be necessary to examine the association not only from social and complex perspectives but also from the classical and primitive perspectives of emotional valence (positive or negative). Therefore, it is important to examine the association between emotional valence and nonverbal channels. Moreover, it is possible to perceive positive emotions more effectively through touch than through other channels. Because touch itself can have positive consequences (Crusco & Wetzel, 1984; Fisher et al., 1976), and only intimate persons are normally allowed to touch the other's body in daily communication (Suvilehto et al., 2015; Suvilehto et al., 2019), people may be positively biased in the intended emotion of those touching.

Additionally, the number of emotional categories differed among studies examining different channels; this leads to different chance levels among the studies. Oya and Tanaka (2022) revealed that the accuracy of emotion perception from touch was lower than that of faces and voices. It may be because the number of response alternatives and chance levels varied between channels (see discussion in Oya & Tanaka, 2022). To compare channels appropriately, we should match the number of emotional categories between channels.

The second factor that makes it difficult to compare channels is the method used to present stimuli. Previous studies have used different methods for different channels. The lower accuracies observed in previous studies on touch may be attributable to the different methods employed in the research on face, voice, and touch. Most studies on the perception of emotions through face and voice have recorded and controlled stimuli. In contrast, studies on touch use the free expression method, because it is not possible to record and present the same physical contact. These studies employed a method in which the encoder freely expressed emotions by touching the decoder's arm or body (Hertenstein et al., 2006, 2009; Oya & Tanaka, 2022). This method may lead to lower accuracy than a method that uses recorded and controlled stimuli. To determine whether lower accuracy is specific to emotion perception from touch or due to different presentation methods, it is necessary to compare emotion perception from touch and other channels using the free expression method, which has been used previously in studies on touch.

This study investigated whether different channels (touch and voice) have different advantages for the perception of positive and negative emotions. We chose vocal expressions rather than facial expressions for comparison with touch because several positive emotions other than happiness can be perceived from voice. It should be noted that several studies have repeatedly shown that vocal expressions can discriminate between positive emotion categories other than happiness, such as amusement, pleasure, relief, and triumph (e.g., Simon-Thomas et al., 2009), although recent research has also reported that various positive emotions can be perceived from facial expressions (Cowen & Keltner, 2020). We adopted the same paradigm used in previous research on touch. In the experiment, the encoder freely expressed basic, self-focused, and prosocial emotions by touching the decoder's arm or by uttering /e/. The utterance /e/ is often used as an interjection in Japanese with affective prosody (Arimoto & Okanoya, 2011). Therefore, chance level and methodology were matched for touch and voice. We examined whether the decoder could perceive the expressed emotion and compared the advantages of touch and voice in the perception of positive and negative emotions. We hypothesize that touch has an advantage over voice in the perception of positive emotions because touch is normally used in a positive situation and/or between intimate relationships.

## Method

### Participants

Fifty-four Japanese women (*M* = 20.33 years, *SD* = 1.16) participated in the study. They provided verbal consent, and the study was approved by the Ethics Committee of Tokyo Woman's Christian University.

The participants were randomly assigned to the dyads. One member of each dyad was assigned the role of touching (encoder, *n* = 27), and the other member was assigned the role of being touched (decoder, *n* = 27). The participants did not have any information about their partners.

We conducted a post-hoc power analysis for our sample size (27 dyads) using the Pangea online power calculator. The result indicated 0.95 power to detect Channel (touch and voice) × Emotional valence (positive and negative) interaction of *d* = .45.

### Apparatus

A desk (RAC-EC2SN, Sanwa Supply) was divided into two sections using a black curtain. The encoder and decoder were seated on opposite sides of the desk separated by a curtain. A video camera (HDR-PJ540, Sony) mounted on a tripod (TH-650DV, Libec) was placed on the side of the encoder to record the display of the encoder.

### Procedure

The experiment consisted of two sessions: a touch session and a voice session. The procedures used in the current study were nearly identical to that of Hertenstein et al. (2006) for the “touch session” and slightly modified for the “voice session.” We conducted 12 trials in each session (anger, fear, happiness, sadness, disgust, surprise, embarrassment, envy, pride, love, gratitude, and sympathy) for a total of 24 trials. The order of the sessions and trials was counterbalanced.

**Touch session*.*** Initially, the encoder practiced on a hand mannequin in the laboratory by expressing each emotion once. After the practice session, the experimenter provided the following instructions to the encoder: First, the encoder can touch the decoder's arm (including the hand) as far as they can see from behind the curtain. Second, the encoder should touch in any way that they thought appropriate if it did not hurt the decoder. Third, the encoder cannot speak until the end of the experiment. After this, the experimenter entered another room and requested that the decoder not speak until the end of the experiment. The decoder then enters the laboratory. When the experiment started, the decoder extended their arm to the encoder's area. The encoder then touched the decoder's arm and attempted to convey the instructed emotion through touch. Once the encoder finished, the experimenter handed out a response sheet to the decoder. The decoder indicated the perceived emotion by selecting one of the 13 response options, which included the names of the 12 emotions and a statement, “None of these terms are correct.” The instructions in the response sheet stated, “Please choose the term that best describes what this person is communicating to you.” The placement of the 12 emotions on the response sheet was randomized across participants. After the response sheet was recovered, the next trial began. This process was repeated 12 times.

**Voice session*.*** The voice session was conducted using almost the same procedure as the touch session but with a different method of expressing emotions. In a voice session, the encoder practiced expressing each emotion once by uttering /e/, which is often used as an interjection in Japanese, with affective prosody (Arimoto & Okanoya, 2011). The experimenter instructed the encoder that they could utter/e/ in any way that they thought appropriate. After the practice session, the experiment was initiated. During the experiment, the encoder expressed each of the 12 emotions by uttering /e/. Once the encoder finished the utterance, the experimenter handed out a response sheet to the decoder, who answered in the same manner as in the touch session.

### Experimental Design

The emotional valence (positive and negative) of an emotion expressed by the encoder and the nonverbal channel (touch and voice) used in each session served as independent variables, and the accuracies served as the dependent variables.

During the experiment, the encoder simultaneously expressed each emotion. Six of 12 emotions (anger, fear, sadness, disgust, embarrassment, and envy) had negative valence, and five emotions (happiness, pride, love, gratitude, and sympathy) had positive valence. Therefore, the conditions of negative and positive valence were repeated six and five times in each session, respectively.

### Coding Procedure

Based on the video movies recorded in the touch session, two coders independently coded all encoders’ tactile expressions as action categories using the same methods as in Oya and Tanaka (2022). Data from 26 encoders were coded (we could not record one movie session owing to technical problems). Thirteen categories were used to code the encoders’ actions: *osu* (pushing, pressing); *nigiru* (squeezing); *sasuru/naderu* (stroking, rubbing); *oshinokeru/mukouniyaru* (poking); *yowaku-tataku* (patting, tapping); *tsuyoku-tataku*(hitting, slapping); *tsuneru* (picking, pinching); *furu/yurasu* (swinging, shaking); *kusuguru* (tickling); *furuesaseru* (trembling); *mochiageru* (lifting); *tsutsumu* (overlapping); *soeru* (putting on).

### Data Analysis

To examine whether the decoder could perceive the encoder's expressed emotion as categorical, we calculated the categorical accuracy showing the degree to which the decoder's response (perceived emotion) was congruent with the emotion expressed by the encoder (expressed emotion). To examine whether emotions can be perceived from each channel, a one-sample *t*-test was conducted on the average accuracy of the 12 emotions on each channel. Thereafter, to examine whether each channel was suited to perceive positive and negative emotions, an analysis of variance (ANOVA) was conducted for Emotional valence (positive or negative) × Channel (touch or voice) on the average accuracy in each condition. Finally, confusion matrices were constructed to reveal channel-specific confusions in emotional valence. The data were analyzed using IBM SPSS Statistics (Version 27).

## Results and Discussion

### Emotions Were Perceived From Both Channels

Table 1 shows the confusion matrices that contain both categorical accuracies (underlined) and confusions. To examine whether the decoders could perceive emotions through each channel, we averaged the 12 categorical accuracies for each channel within each subject and conducted one-sample t-tests for the average of touch and voice. We set the chance level at 7.69% because the decoders judged the expressed emotion from 13 categories. The results showed that both the average accuracy of touch (*t*[26] = 6.35, *p *< .001, 95% CI [0.11, 0.22]) and that of voice (*t*[26] = 8.70, *p *< .001, 95% CI [0.16, 0.26]) were significantly higher than the chance. Therefore, emotions were perceived above the chance level from both touch and voice.

**Table 1.** The Confusion Matrices Contain Both Categorical Accuracies (Underlined) and Confusions.

(A)													
*PE*													
*EE*	AN	FE	SA	DI	EM	EN	SU	HA	PR	LO	GR	SY	None
AN	37.0	7.4		22.2	7.4	7.4	11.1			3.7		3.7	
FE	7.4	11.1	7.4		11.1	7.4	7.4	3.7		7.4	11.1	14.8	11.1
SA		11.1	11.1	3.7	14.8	7.4			3.7	18.5	7.4	18.5	3.7
DI	25.9		7.4	25.9	7.4	14.8	3.7	3.7	3.7			3.7	3.7
EM		7.4	18.5	3.7	29.6	3.7			3.7	7.4	11.1	7.4	7.4
EN	14.8	3.7		25.9	3.7	25.9			3.7	7.4	11.1	3.7	
SU	11.1		7.4	22.2	7.4	3.7	11.1	11.1	3.7	7.4		14.8	
HA	3.7						3.7	40.7		7.4	18.5	18.5	7.4
PR		3.7	3.7	3.7	7.4		3.7	14.8		11.1	29.6	14.8	7.4
LO							3.7	3.7	3.7	37.0	25.9	22.2	3.7
GR	3.7							18.5	7.4	11.1	48.1	11.1	
SY			3.7					7.4	3.7	40.7	25.9	14.8	3.7
*M*	14.8	7.4	8.5	15.3	11.1	10.1	6.3	13.0	4.2	14.5	21.0	12.3	6.0

(B)													
*PE*													
*EE*	AN	FE	SA	DI	EM	EN	SU	HA	PR	LO	GR	SY	None
AN	33.3			29.6	11.1	7.4	14.8	3.7					
FE		14.8	11.1	3.7	22.2		33.3	7.4					7.4
SA		3.7	51.9	11.1	14.8	7.4	3.7	3.7					3.7
DI	7.4		11.1	51.9	22.2	3.7							3.7
EM		3.7	14.8	29.6	37.0		11.1						3.7
EN	11.1		7.4	33.3	14.8	7.4	7.4				3.7	11.1	3.7
SU	3.7	3.7			7.4	3.7	70.4	7.4			3.7		
HA	3.7			7.4	3.7		22.2	44.4			7.4	11.1	
PR				11.1	14.8	3.7	25.9	18.5	3.7			7.4	14.8
LO			7.4	11.1	22.2	3.7	22.2	7.4		3.7		18.5	3.7
GR		7.4	22.2	7.4	7.4		3.7	22.2				25.9	3.7
SY	3.7		3.7	11.1	25.9		11.1	7.4		3.7		29.6	3.7
*M*	10.5	6.7	16.2	18.9	17.0	5.3	20.5	13.6	3.7	3.7	4.9	17.3	5.3

*Note*. Table 1A and B shows the confusion during the touch and voice sessions, respectively. The labels of emotions refer below: EE = expressed emotion, PE = perceived emotion, AN = anger, FE = fear, SA = sadness, DI = disgust, EM = embarrassment, EN = envy, SU = surprise, HA = happiness, PR = pride, LO = love, GR = gratitude, and SY = sympathy. “None” refers to the response, “None of these terms are correct.” Null cells indicate that emotion was not perceived when it was expressed. Underbars indicate correct responses (i.e., categorical accuracy). Both Table 1A and B are colored in heatmap format; the higher the response rate, the darker the color of the cell. Responses judged as positive are orange, and those judged as negative are indicated in blue.

### Different Channels Have Different Advantages in the Perception of Positive and Negative Emotions

**Figure 1.**
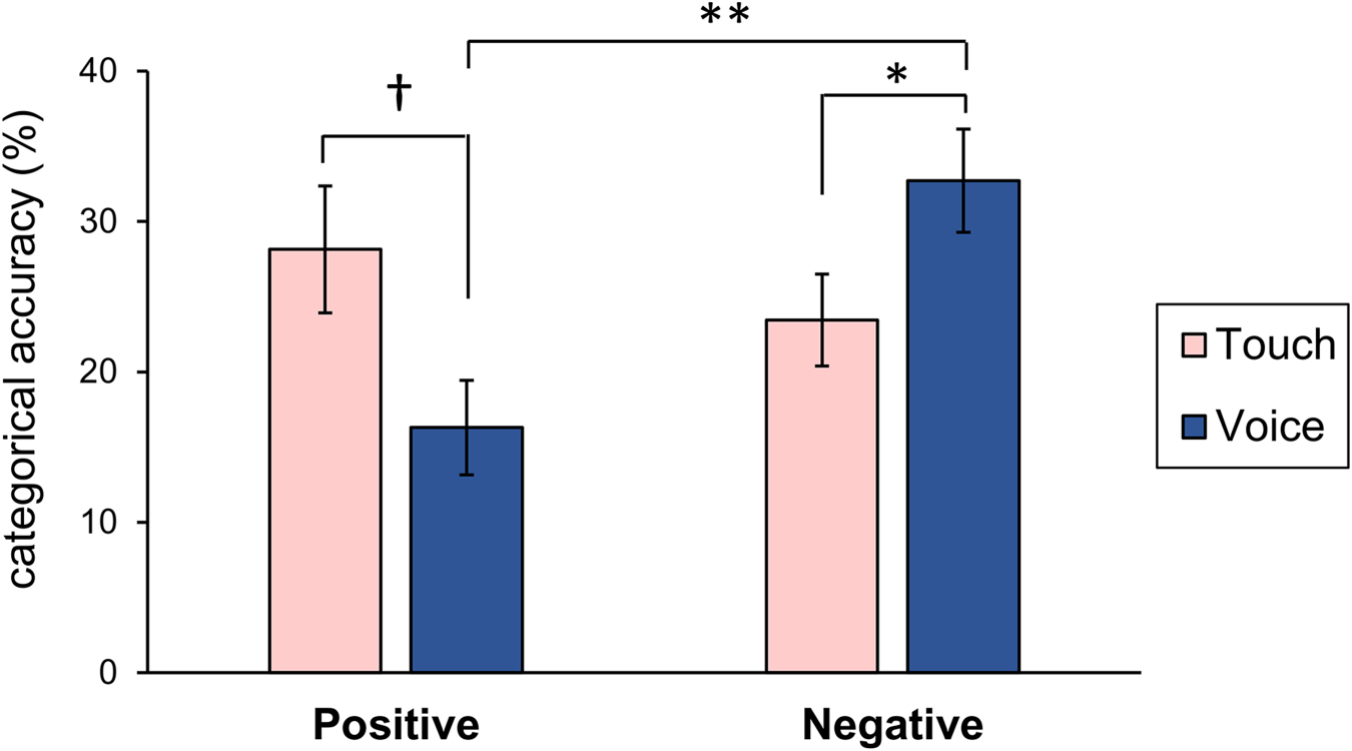
Mean accuracy of touch and voice conditions in positive and negative emotions.

*Note.* Error bars represent standard error. Asterisks indicate significant differences (**p < .01, *p < .05, ^†^*p *< .10).

To examine whether different channels convey different emotional valences, 11 of the 12 emotions examined in this study (excluding surprise, which has both positive and negative valences) were divided into positive (happiness, pride, love, gratitude, and sympathy) and negative (anger, fear, sadness, disgust, embarrassment, and envy) emotions. We then averaged the categorical accuracy for positive and negative emotions and conducted an ANOVA of the expressed emotional valence (positive or negative) × channel (touch or voice) within the participants (Figure 1).

Although the main effect of the channel was not significant (*F*[1, 26] = 0.15, *p* = .70, *η*²_p_ = .01), the main effect of emotional valence was marginally significant (*F*[1, 26] = 3.93, *p *< .10, *η*²_p_ = .13). Importantly, the two-way interaction between emotional valence and the channel (*F*[1, 26] = 7.40, *p *< .05, *η*²_p_ = .22) was significant. Simple main effect analyses showed that the average accuracy of positive emotions was marginally higher for touch than for voice (*F*[1, 26] = 3.94, *p *< .10, 95% CI [0.00, 0.24]), whereas that of negative emotions was higher for voice than for touch (*F*[1, 26] = 5.08, *p *< .05, 95% CI [0.01, 0.18]). Thus, the results suggest that voice has an advantage for negative emotions, while touch does not have a negative advantage and shows a marginal advantage for positive emotions, partially supporting our hypothesis.

### Channel-Specific Confusion

Table 1 also shows the confusion in the touch (Table 1A) and voice sessions (Table 1B). To examine how the decoder confused the expressed emotions at the emotional valence level, we counted the frequency of confusion for each participant. Specifically, we divided confusion into four patterns: positive emotions were perceived as positive (PP), positive emotions as negative (PN), negative emotions as positive (NP), and negative emotions as negative (NN). PP and NN were within-valence confusions, and PN and NP were between-valence confusions. Then, we counted the frequency of PP, PN, NP, and NN for each participant and constructed a cross table of expressed emotional valence (positive or negative) and perceived emotional valence (positive or negative) for the confused trials. The Chi-squared test for the cross table was significant for both touch (*χ*^2^ [1] = 56.6, *p *< .001) and voice (*χ*^2^ [1] = 23.27, *p *< .001).

To further examine how confusion was biased in each channel, we conducted additional analyses. These results provide two important suggestions. First, when the encoder expressed positive and negative emotions, the decoder tended to perceive positive and negative valences, respectively. We conducted a residual analysis for the cross table of each channel, which examined whether the frequency of each cell was significantly higher than expected. The results showed that the frequencies of PP and NN were significantly higher than expected, whereas those of PN and NP were lower than expected. These results suggest that most of the confusion was observed in emotional valence.

Second, we conducted *t*-tests between the channels for the frequencies of PP, PN, NP, and NN to reveal channel-specific confusion. The results showed that NP confusion was more frequently observed in the touch session than in the voice session (*t*[26] = 4.98, *p *< .001, 95% CI [0.76, 1.83]), whereas PN confusion was more frequently observed in voice sessions than in touch sessions (*t*[26] = −5.74, *p *< .001, 95% CI [−2.01, −0.95]). The results showed that touch biased the perception of emotions toward positive and voice biased the perception toward negative.

### Action of Touch

Although it is difficult to compare the results with those of Hertenstein et al. (2006) because of the differences in coding categories, the actions were similar to those of previous studies for some emotions (e.g., anger and happiness), but not for others (e.g., sadness and love). To examine cross-cultural similarities in the actions, we conducted a correlation analysis on the proportion of people who showed the three most frequent types of touch in Hertenstein et al. (2006) between studies (for details, see Oya & Tanaka, 2022). There was a positive correlation between Hertenstein et al. (2006) and the current study (*r* = .49, *p *< .01), suggesting that the actions of the encoder in this study were similar across cultures. Furthermore, to examine whether tactile behavior is similar within cultures, we conducted a correlation analysis between Oya and Tanaka (2022) and the current study; there was a strong positive correlation between the studies (*r* = .89, *p *< .01), suggesting that the tactile action of emotional expressions was also similar within cultures.

## General Discussion

### Summary of Results

In this study, we investigated whether different channels (touch and voice) have different advantages in terms of the perception of positive and negative emotions. In the experiment, the encoder freely expressed 12 emotions by touching the decoder's arm or uttering /e/. The decoder was asked to judge the emotions expressed. The chance levels and methodologies for touch and voice were matched.

The results revealed that voice has an advantage for negative emotions, while touch does not have a negative advantage and shows a marginal advantage for positive emotions. Therefore, our results suggest that different channels are advantageous in terms of the perception of positive and negative emotions. These results are consistent with previous research suggesting that different emotions are associated with different nonverbal channels: the body, face, and touch (App et al., 2011).

### One Possible Interpretation of Our Results

We speculate that these results can be explained in terms of approach-avoidance motivation (Elliot et al., 2013). Generally, when the approach-avoidance motivation between the expressed channel (touch or voice) and emotional valence (positive or negative) matches, the expressed emotion is perceived more easily. Regarding the channel, touch is a channel of approach because touching requires direct contact with the partner's body, whereas voice does not (Schirmer & Adolphs, 2017). The voice is a channel of avoidance because it is useful for quickly noticing danger. Regarding emotions, positive emotions tend to have an approaching motivation because they increase the benefit for others, and negative emotions tend to have an avoidance motivation because they often function to escape from others (for a similar discussion, see Watson et al., 1999). Assuming these correspondences, we speculate channel-valence associations as follows: positive emotions are perceived more accurately from touch than from voice because of approach-motivation, and negative emotions from voice than from touch because of avoidance-motivation.

However, the results indicated that touch showed only a marginal advantage for positive emotions. We should note the motivation of anger: anger is a negative emotion but an approach-oriented motivation (Carver & Harmon-Jones, 2009). Several studies have revealed that anger can be perceived from touch (e.g., Hertenstein et al., 2006; Oya & Tanaka, 2022), suggesting another association: touch may have an advantage in the perception of approach-oriented emotion.

### Alternative Perspective: Functional Role of Emotions

In this study, we revealed a channel-valence association. Emotional valence is one of the core and primitive frameworks of emotions. However, this distinction—positive versus negative—is not the only criterion. Previous research has revealed that distinct emotions of the same valence may have different effects on judgment (Lerner & Keltner, 2000). Therefore, it is also possible that our data suggest an association between the channel and other emotion classifications.

An alternative framework has been widely proposed, and one of the candidates is based on the social function of emotions (e.g., Keltner & Haidt, 1999). For instance, Elfenbein et al. (2007) distinguished emotions mainly elicited in social interaction from those that are often elicited by non-social events. Based on their argument, we assumed that anger, happiness, love, and gratitude are more socially relevant emotions (for the social function of love and gratitude, see Keltner & Kring, 1998). In contrast, disgust and surprise are supposed to be more non-social or self-relevant. Given this classification, we speculate that the channel-social function association is also possible: socially relevant emotions such as anger (accuracy of touch:37.0, voice:33.3), love (touch:37.0, voice:3.7), and gratitude (touch:48.1, voice:0.0) can be perceived more correctly from touch, and self-relevant emotions such as disgust (touch:25.9, voice:51.9) and surprise (touch:11.1, voice:70.4) from voice. Thus, future research should examine channel preferences from multiple perspectives, including the social functions of emotions.

### Comparison With Previous Research on the Emotion Perception From Touch

In this study, only gratitude was perceived to be above the conservative chance level of previous touch studies (i.e., 25%; Hertenstein et al., 2006; Oya & Tanaka, 2022) in touch sessions. Fear, disgust, and sympathy were not perceived through touch, thus replicating the cultural specificity reported by Oya and Tanaka (2022). However, the accuracy of anger (37.0%) and love (37.0%) was not significantly higher than that of conservative chance in this study, whereas these emotions were perceived above chance (anger: 37.7%; love: 43.4%) in Oya and Tanaka (2022). Although the categorical accuracies were almost comparable between studies, the current study could not detect statistical significance owing to the sample size design. In Oya and Tanaka (2022), the sample size was 53 (dyads) to detect whether each emotion was perceived (i.e., categorical accuracy above conservative chance). However, the current study optimized the sample size to detect the channel-valence association (i.e., the two-way interaction between the emotional valence and channel). Consequently, our sample size was insufficient to detect whether each emotion was perceived above the conservative chance level.

In this study, negative emotions (average of six negative emotions: 23.4%) were perceived less accurately by touch, compared to Hertenstein et al. (2006). We could point out at least three possible factors that could influence the results of negative emotions: tactile behavior, culture, and gender. First, analyses of tactile actions showed that the tactile actions of emotional expressions were similar within and between cultures. Therefore, it is not plausible that tactile behavior could explain the differences between Hertenstein et al. (2006) and our results. Second, fear and disgust were perceived through touch in the United States and Spain (Hertenstein et al., 2006), but not in Japan (Oya & Tanaka, 2022). These two emotions were not perceived from touch in either Oya and Tanaka (2022) or the current study, suggesting cultural specificity in the perception of these emotions from touch. Third, there might also be gender differences, because our experiment employed only female participants. Hertenstein and Keltner (2011) reported gender differences in the perception of emotions through touch (the accuracy of anger was 37.5% between female dyads and 70.4% between male dyads). The accuracy between female dyads was comparable to that of the current study (37.0%), suggesting that the low accuracy for anger is due to gender differences. Taken together, it is plausible that lower accuracies for negative emotions were due to cultural and/or gender differences in the perception of emotions from touch.

### Limitations and Future Directions

This study had some limitations. First, the encoder expressed emotions using only one type of syllable, /e/, in a voice session, whereas they expressed emotions in various ways in the touch session. Previous studies regarding the perception of emotions from voice have used two different types of vocalizations: emotional prosody and vocal burst. Regarding emotional prosody, the expresser encodes emotions using a vocal tone with a semantically neutral sentence (e.g., “*What is it?*”). As for vocal bursts, the expresser encodes emotions without using any word and produces brief non-linguistic sounds (e.g., laughing and crying). In the voice session, the decoders’ identifications converged for certain emotions, such as disgust and surprise. This tendency was not observed in touch sessions or other research using speech prosody. Thus, channel preference may differ depending on how the encoder is instructed to express emotions through voice. Therefore, future research should compare emotion perception between speech prosody with neutral sentences and vocal bursts.

Second, only females participated in this study to enable a comparison with Oya and Tanaka (2022). However, Hertenstein and Keltner (2011) reported gender differences in emotion perception from touch in the United States, which might also be the case in Japan. Future studies should consider gender differences by including both male and female participants.

The third limitation was the sample size. Our post-hoc power analysis indicated sufficient power to detect channel-valence interactions. However, the sample size should be justified before data collection, for instance doing an a-priori power analysis (Lakens, 2022). Future studies should carefully justify that the sample size is more informative.

Interestingly, the results also showed that accuracy did not significantly differ between touch and voice. It is possible to interpret these results from two opposing perspectives. On the one hand, the accuracies of both channels may be essentially equal. Oya and Tanaka (2022) pointed out that the accuracy of touch is apparently lower than that of the face and voice. The authors argued that the lower accuracy of touch might be due to the difference in the chance level and/or that of the presentation method between touch and other channels. In this study, we employ the same method across channels. As a result, the accuracy was almost comparable between the channels, suggesting that emotions can be perceived from both touch and voice to the same extent. However, the accuracies of the two channels may be different. It is possible that the results reflect artifacts from the free expression method and emotional categories. As for the method, we adopted the free expression method because it is not possible to record and present the same physical contact repeatedly. Although it is difficult to record interpersonal touch and present the same tactile stimuli repeatedly, future research will need to compare various nonverbal channels under controlling emotional stimuli. Tactile representations are composed of multiple parameters, including location, action, and duration (Hertenstein, 2002). One possible solution is to control one of them systematically. Next, for the emotional categories, we applied the emotional categories used in touch studies to the voice. Thus, it might be difficult to perceive the positive emotions used in the current study from voice. Note that the accuracy did not differ between touch and voice in positive valence, although it did differ significantly in negative valence. A previous study showed that vocal expressions can discriminate among positive emotion categories other than happiness, such as amusement, pleasure, relief, and triumph, whereas facial expressions cannot (Sauter, 2010). Based on the literature, the accuracy of perceiving positive emotions from voice may be comparable to that of touch. In other words, it also remains possible that both touch and voice are suited to perceiving positive valence, although each channel prefers different positive emotions. Future research should use additional emotional categories, especially the positive emotions employed in voice studies, and compare superiority in the perception of positive and negative emotions among channels.
